# *Indigo Pulverata Levis* (Chung-Dae, *Persicaria tinctoria*) Alleviates Atopic Dermatitis-like Inflammatory Responses In Vivo and In Vitro

**DOI:** 10.3390/ijms23010553

**Published:** 2022-01-05

**Authors:** Ga-Yul Min, Ji-Hye Kim, Tae-In Kim, Won-Kyung Cho, Ju-Hye Yang, Jin-Yeul Ma

**Affiliations:** Korean Medicine (KM) Application Center, Korea Institute of Oriental Medicine, 70 Cheomdan-ro, Dong-gu, Daegu 41062, Korea; gayul8955@kiom.re.kr (G.-Y.M.); jkim2903@kiom.re.kr (J.-H.K.); tikim@kiom.re.kr (T.-I.K.); wkcho@kiom.re.kr (W.-K.C.)

**Keywords:** atopic dermatitis, *Indigo Pulverata Levis*, immune-cell infiltration, skin thickness, NF-κB p65, proinflammatory cytokines

## Abstract

Atopic dermatitis (AD) is a chronic inflammatory skin disease associated with a type 2 T helper cell (Th2) immune response. The *Indigo* *Pulverata Levis* extract (CHD) is used in traditional Southeast Asian medicine; however, its beneficial effects on AD remain uninvestigated. Therefore, we investigated the therapeutic effects of CHD in 2,4-dinitrochlorobenzene (DNCB)-induced BALB/c mice and tumor necrosis factor (TNF)-α- and interferon gamma (IFN)-γ-stimulated HaCaT cells. We evaluated immune cell infiltration, skin thickness, and the serum IgE and TNF-α levels in DNCB-induced AD mice. Moreover, we measured the expression levels of pro-inflammatory cytokines, mitogen-activated protein kinase (MAPK), and the nuclear factor-kappa B (NF-κB) in the mice dorsal skin. We also studied the effect of CHD on the translocation of NF-κB p65 and inflammatory chemokines in HaCaT cells. Our in vivo results revealed that CHD reduced the dermis and epidermis thicknesses and inhibited immune cell infiltration. Furthermore, it suppressed the proinflammatory cytokine expression and MAPK and NF-κB phosphorylations in the skin tissue and decreased serum IgE and TNF-α levels. In vitro results indicated that CHD downregulated inflammatory chemokines and blocked NF-κB p65 translocation. Thus, we deduced that CHD is a potential drug candidate for AD treatment.

## 1. Introduction

Atopic dermatitis (AD) is a chronic inflammatory skin disease caused by an imbalance in the immune response [1]. Notably, AD has a complex etiology, including environmental and genetic factors, and is a public health problem as it affects 15–20% of children and 2–4% of adults worldwide [2]. Moreover, it causes asthma and allergic rhinitis and is characterized by symptoms such as erythema, rash, edema, dryness, and skin hypersensitivity [3,4]. Owing to these characteristics, AD patients suffer from sleep disturbances, school performance problems, anxiety, stress, and lower life quality [5]. Immunosuppressants, antihistamines, and corticosteroids have been widely used for the treatment of AD [6]. However, their long-term use causes side effects, such as skin atrophy, emotional instability, and exacerbated bacterial/viral skin infections [7]. Thus, natural products as alternative medicines are gaining interest, as they balance the immune system and are safe [8].

The mechanisms underlying AD development include thickening of the dermis and epidermis, increasing serum levels of immunoglobulin E (IgE), and invasion of immune cells (e.g., eosinophils and mast cells) into the dermis [9]. However, the pathological mechanism of acute AD involves skin inflammation primarily caused by T helper (Th) 2 cell signaling. In contrast, Th2 signaling is converted to Th1 signaling in the chronic condition [10]. Upregulated Th2 signaling stimulates B cells and increases IgE secretion, leading to the degranulation of dermis mast cells and expression of inflammatory cytokines and chemokines [11]. This causes allergic symptoms that exacerbate AD [12]. Extracellular signal-regulated kinase (ERK), c-jun N-terminal kinase (JNK), and p38 that have been implicated in the inflammatory signaling cascade play an important role in differentiation of inflammatory cells and inhibition of allergic inflammation [13]. The transcription factor NF-κB is also a crucial regulatory factor of inflammatory mediators [14]. Therefore, activation of these signaling pathways promotes the synthesis of inflammatory cytokines and chemokines [15].

*Indigo Pulverata Levis* (*Persicaria tinctoria*), known as “Chung-Dae” (CHD), is a blue powder extracted from the indigo plant. [16]. Of note, CHD has been used for centuries in traditional oriental medicine to treat infectious, inflammatory diseases, ulcerative colitis, and latent fever [17,18]. Previously, studies have reported that CHD has antipyretic, anti-inflammatory, and detoxifying properties. Recently, it has been demonstrated to have excellent clinical efficacy in treating gastrointestinal diseases and AD [19,20,21]. Furthermore, CHD contains indigo and indirubin that are effective in treating various diseases, such as bacterial infections, cancer, and inflammation, by affecting the immune system [22,23]. However, the detailed mechanism of action of CHD in treating AD remains unclear.

In this study, we evaluate the anti-AD effect of CHD in the human keratinocyte cell line HaCaT and in 2,4-dinitrochlorobenzene (DNCB)-induced AD animal models. We investigated the effect of CHD on epidermis and dermis thicknesses and mast cell and eosinophil infiltration in AD mice. In addition, we measured the expression levels of IgE and tumor necrosis factor alpha (TNF-α) in the serum and that of inflammatory cytokines, nuclear factor-kappa B (NF-κB), and mitogen-activated protein kinase (MAPK) in the dorsal tissue. We also elucidated the mechanism of inhibition of AD by CHD and investigated the expression of chemokines and translocation of NF-κB p65 in TNF-α- and IFN-γ-stimulated HaCaT cells. Eventually, we integrated all our results to determine the potential applicability of CHD for the treatment of AD. We also conducted HPLC analysis to identify the compounds in CHD, and the contents were confirmed.

## 2. Results

### 2.1. Effects of CHD on AD Symptoms in Mice

**Figure 1:**
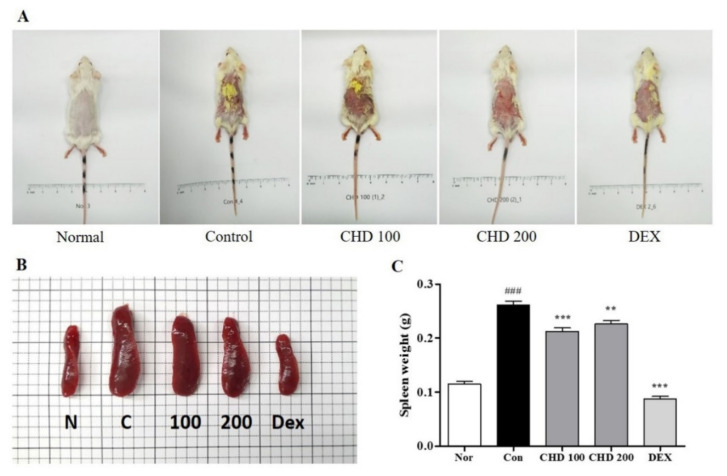
Effects of the *Indigo Pelvarata Levis* extract (CHD) on dorsal skin lesions and spleen hypertrophy in mice with atopic dermatitis (AD). (**A**,**B**) Photographs of dorsal skin lesions and spleens in AD mice. (**C**) Measurement of spleen hypertrophy. Data represent the mean ± standard error of the mean. *n* = 10. ^###^ *p* < 0.001 vs. normal group; ** *p* < 0.01 and *** *p* < 0.001 vs. DNCB-induced group.

We determined the therapeutic effects of CHD in the DNCB-induced BALB/c mice models. As shown in Figure 1A, the control group had erythema, edema, and eczematous skin lesions, whereas the CHD-treated groups exhibited less severe AD symptoms. We determined the status of the immune systems in the AD mice by weighing their spleens (Figure 1B). We observed that the spleen weights were greater in the DNCB-induced control group than those in the normal group. However, the CHD treatment groups exhibited a significant decrease in the spleen weights compared to the control group (Figure 1C).

### 2.2. Effects of CHD on Epidermis and Dermis Thicknesses in AD Mice

**Figure 2:**
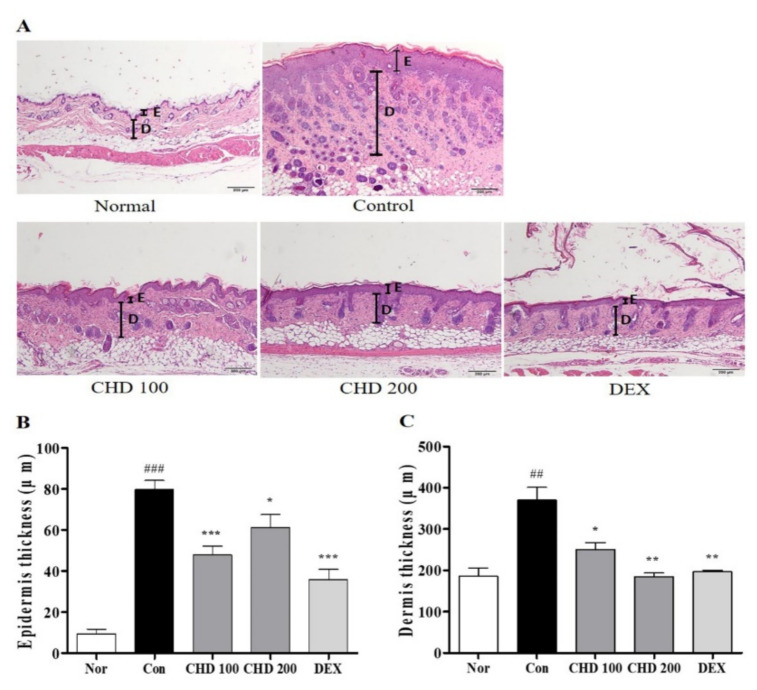
Effects of the *Indigo Pelvarata Levis* extract (CHD) on the histological characteristics of atopic dermatitis mice models. (**A**) Epidermis and dermis thicknesses were examined by H&E staining (100× magnification; scale bar: 200 µm). (**B**,**C**) Measurement of epidermis and dermis thicknesses. Data represent the mean ± standard error of the mean. *n* = 10. ^##^ *p* < 0.001 and ^###^ *p* < 0.01 vs. the normal group; * *p* < 0.05, ** *p* < 0.01, and *** *p* < 0.001 vs. the DNCB-induced control group.

We evaluated the effect of CHD on the histological characteristics of the AD mice by staining their dorsal skins with the hematoxylin and eosin (H&E) stain (Figure 2A). The epidermis and dermis thicknesses in the DNCB-induced control group were greater than those in the normal group. In fact, these thicknesses were significantly reduced in the CHD group compared to those in the control group (Figure 2B,C).

### 2.3. Effects of CHD on Immune Cell Infiltration in AD Mice

**Figure 3:**
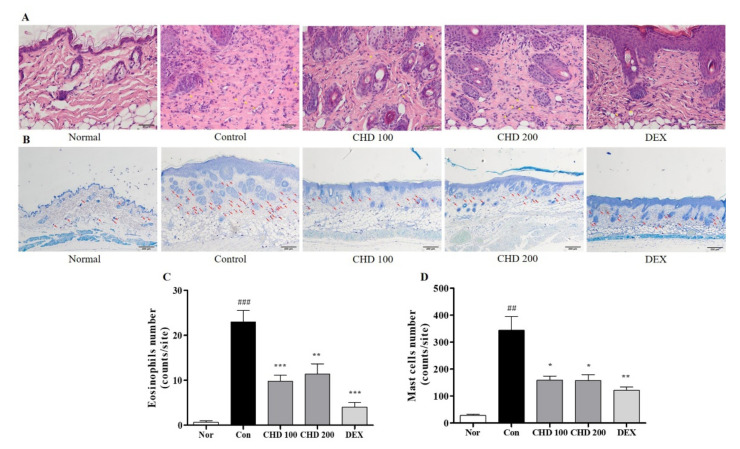
Effects of the *Indigo Pelvarata Levis* extract (CHD) on immune cell infiltration in atopic dermatitis mice models. (**A**,**B**) H&E and toluidine blue stained eosinophils and mast cells that infiltrate into dermis lesions (H&E: 400× magnification, 50 µm scale bar; toluidine blue: 100× magnification, 200 µm scale bar). (**C**,**D**) The number of infiltrating immune cells quantified using the ImageJ software. Data represent the mean ± standard error of the mean. *n* = 10. ^##^ *p* < 0.001 and ^###^ *p* < 0.01 vs. the normal group; * *p* < 0.05, ** *p* < 0.01, and *** *p* < 0.001 vs. the DNCB-induced control group.

We investigated the effects of infiltration of eosinophils and mast cells into the dermis by staining them with H&E and toluidine blue (Figure 3A,B). The DNCB-induced control group had higher numbers of eosinophils and mast cells in the dermis than the normal group. However, the CHD group had significantly low eosinophil and mast cell infiltration compared to the control group (Figure 3C,D).

### 2.4. Effects of CHD on the Levels of IgE and Pro-Inflammatory Cytokines in AD Mice

**Figure 4:**
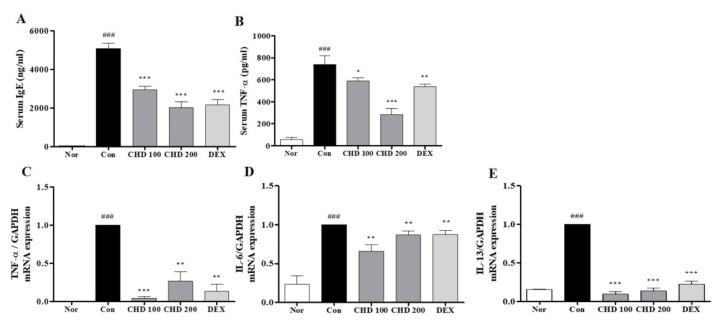
Effects of the *Indigo Pelvarata Levis* extract (CHD) on the IgE and pro-inflammatory cytokine levels in atopic dermatitis mice models. (**A**,**B**) The serum IgE and TNF-α levels were analyzed by the enzyme-linked immunosorbent assay. (**C**–**E**) mRNA expression levels of the cytokines TNF-α, IL-6, and IL-13 were determined by real-time RT-PCR. Data represent the mean ± standard error of the mean. *n* = 10. ^###^ *p* < 0.01 vs. the normal group; * *p* < 0.05, ** *p* < 0.01 and *** *p* < 0.001 vs. the DNCB-induced group.

We performed ELISA and real-time RT-PCR to measure the IgE and TNF-α levels in the serum and the pro-inflammatory cytokine levels in the dorsal mice tissues (Figure 4). The serum IgE and TNF-α levels were significantly higher in the control group than those in the normal group. However, these levels were significantly lower in the CHD-treated groups than those in the control group (Figure 4A,B). In addition, levels of the pro-inflammatory cytokines TNF-α, IL-6, and IL-13 in the dorsal tissue were significantly higher in the control group than those in the normal group. In contrast, these levels were significantly lower in the CHD-treated groups than those in the control group (Figure 4C–E).

### 2.5. Effects of CHD on the MAPK and NF-κB Protein Levels in AD Mice

**Figure 5:**
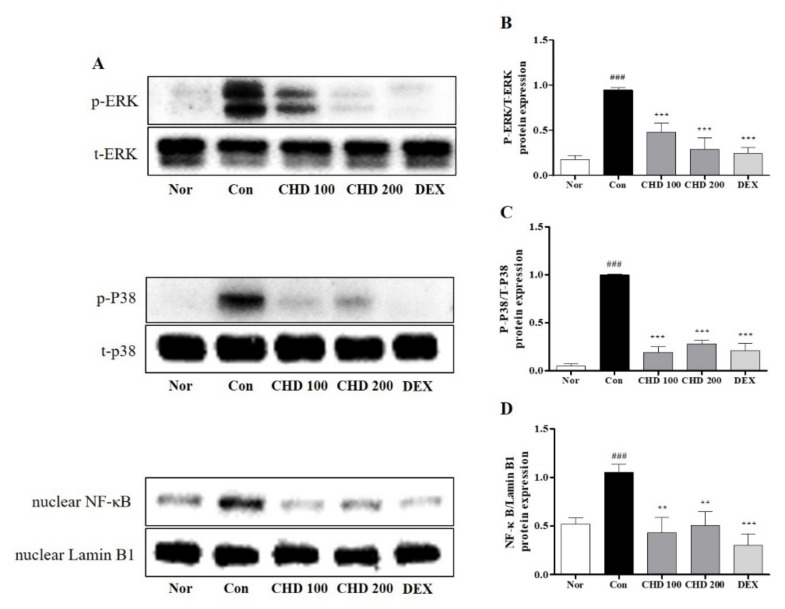
Effects of the *Indigo Pelvarata Levis* extract (CHD) on the MAPK/NF-κB signaling pathway in atopic dermatitis mice models. (**A**) p-ERK, p-p38, and NF-κB expression levels analyzed by Western blotting. (**B**–**D**) Evaluation of the MAPK and NF-κB expression levels using ImageJ. Data are represented as mean ± standard error of the mean. *n* = 5. ^###^ *p* < 0.01 vs. the normal group; ** *p* < 0.01 and *** *p* < 0.001 vs. the DNCB-induced group.

We investigated the anti-inflammatory role of CHD by measuring the protein levels of MAPK and NF-κB and the phosphorylation statuses of ERK and p38 in the mice dorsal skin tissues by Western blotting (Figure 5). While ERK and p38 were phosphorylated in the dorsal tissues of the DNCB-induced control mice, their phosphorylations were significantly inhibited in the CHD-treated groups (Figure 5B,C). Moreover, the nuclear NF-κB gene expression was increased in the DNCB-induced mice dorsal tissues. Notably, the nuclear translocation of NF-κB was inhibited in the CHD-treated groups (Figure 5D).

### 2.6. Effects of CHD on the Chemokine Expression Levels in TNF-α- and IFN-γ-Stimulated HaCaT Cells

**Figure 6:**
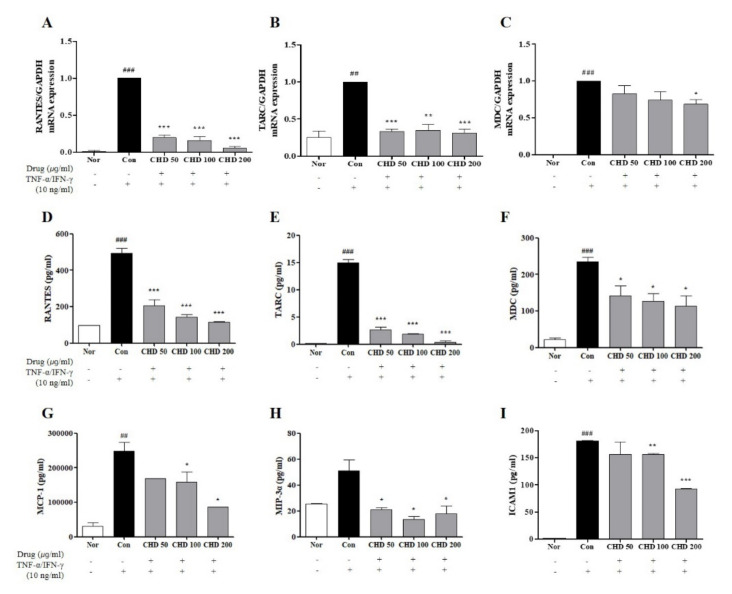
Effects of the *Indigo Pelvarata Levis* extract (CHD) on the chemokine expression levels in HaCaT cells. (**A**–**C**) Production levels of RANTES, TARC, and MDC were determined by real-time RT-PCR. (**D**–**I**) Levels of RANTES, TARC, MDC, MCP-1, MIP-3α, and ICAM1 examined by ELISA. Data represent the mean ± standard error of the mean of three independent experiments. ^##^ *p* < 0.001 and ^###^ *p* < 0.01 vs. the normal group; * *p* < 0.05, ** *p* < 0.01, and *** *p* < 0.001 vs. the control group.

Before entering the in vitro experiment, EZ-Cytox was performed to measure the toxicity of CHD in HaCaT cells. After CHD treatment for 24 hours, it was observed that CHD did not cause cytotoxicity (Supplementary Figure S1). Effects of CHD on the mRNA levels of chemokines were determined by real-time RT-PCR and ELISA. The mRNA expression levels and production of Regulated on Activation, Normal T Expressed and Secreted (RANTES), thymus and activation-regulated chemokine (TARC), and macrophage-derived chemokine (MDC) were higher in the control group than those in the normal group. However, they were significantly lower in the CHD-treated group (Figure 6A–C). In addition, RANTES, TARC, MDC, monocyte chemoattractant protein-1 (MCP-1), macrophage inflammatory protein-3 alpha (MIP-3α), and intercellular adhesion molecule 1 (ICAM1) had higher levels in the cell medium of the control group than those in the normal group. In contrast, their levels were significantly suppressed in the CHD-treated groups (Figure 6D–I).

### 2.7. Effects of CHD on NF-κB p65 Translocation in TNF-α- and IFN-γ -Stimulated HaCaT Cells

**Figure 7:**
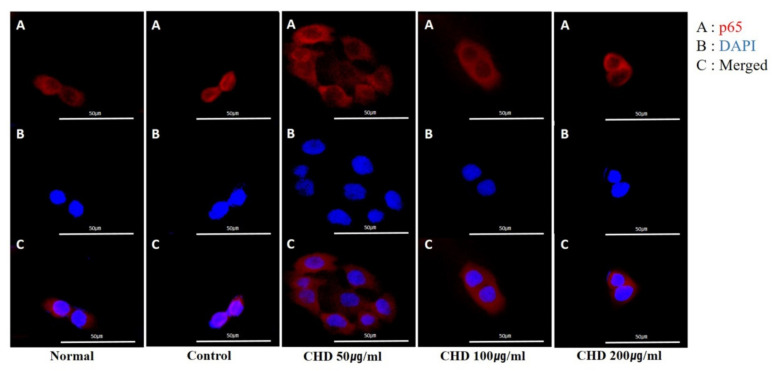
Effects of the *Indigo Pelvarata Levis* extract (CHD) on NF-κB p65 translocation in HaCaT cells. (**A**–**C**) Translocation of p65 was analyzed using a fluorescent microscopy. Representative photomicrographs of (**A**) NF-κB p65 (red), (**B**) DAPI (blue), and (**C**) merged images (nuclear/cytosol) in HaCaT cells.

We determined whether CHD inhibited NF-κB p65 translocation in HaCaT cells stimulated with TNF-α/IFN-γ by immunofluorescence microscopy. Notably, CHD blocked the translocation of NF-κB p65 (Figure 7A–C).

### 2.8. Identification and Quantification of Constituents of CHD

**Figure 8:**
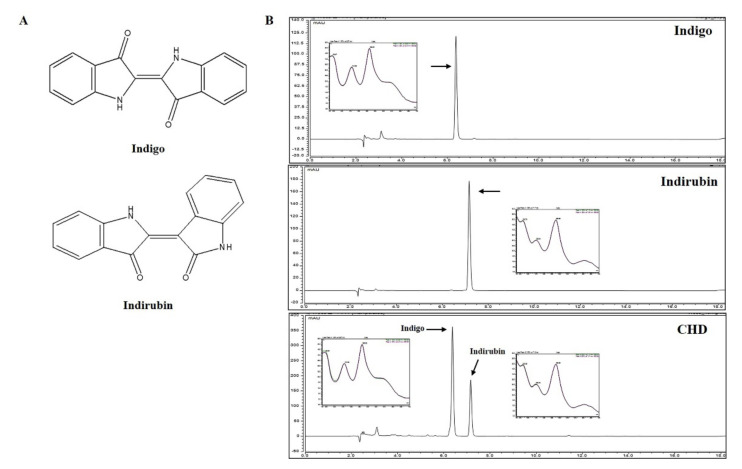
Representative chromatograms of the *Indigo Pelvarata Levis* extract (CHD). (**A**) Chemical structures of indigo and indirubin. (**B**) Standard indigo and indirubin solutions and the CHD extract.

The components of the CHD extract were detected by comparing the chromatograms of CHD with the two marker compounds indigo and indirubin. Indigo and indirubin were selectively detected at 6.370 and 7.160 min; notably, they were also detected in the CHD water extract at the exact same time. Furthermore, the UV chromatograms of the two components and CHD water extract were also analyzed (Figure 8). We validated the contents of the marker compounds in CHD by calculating the area for each component in the CHD chromatogram using a calibration curve of the standard compound at the tested concentration range. The calibration curve of the marker compounds had a good linearity (Table 1). Thus, we determined that the CHD water extract contained 1.18% indigo and 0.10% indirubin.

## 3. Discussion

CHD has been used for centuries to treat infectious, dermatitis, and ulcerative colitis conditions [17,18]. Previously, a study had demonstrated that CHD suppressed AD symptoms in a DNFB-induced NC/Nga mouse model and inhibited thymic stromal lymphopoietin (TSLP) and inflammatory cytokines by blocking the RIP2/caspase-1 signaling pathway in HMC-1 cells [21]. Although there is a similarity in that CHD is effective for Anti-AD by reducing the expression of inflammatory cytokines and chemokines in the skin thickness and dorsal tissue, the symptoms in DNCB-induced BALB/c mice were not confirmed. In addition, inhibition of AD symptoms through inhibition of NF-κB p65 nuclear translocation and chemokine production in HaCaT cells remains unconfirmed. We investigated the effects of CHD on AD symptoms and found that it reduced epidermal and dermis thicknesses and inhibited inflammatory cell infiltration in DNCB-induced BALB/c mice. Notably, it inhibited the expression of IgE and TNF-α in the serum. It also suppressed TNF-α, IL-6, and IL-13 expression levels and MAPK/NF-κB phosphorylation in the dorsal tissues. Furthermore, it reduced inflammatory chemokine production and NF-κB p65 translocation in HaCaT cells. Therefore, we propose that CHD is a potential novel natural alternative for AD treatment.

Thickening of the skin is a major clinical symptom of AD and is caused by the interaction between Th1 and Th2 cells [24]. Persistent allergic inflammatory responses causes infiltration of mast cells and eosinophils in AD lesions by thickening and breaking the epidermis and dermis because of remodeling of the skin surface [25,26]. In a previous study, it was reported that *Naju Jjok* (Chung-Dae, *Polygonum tinctorium*) improved the AD inflammatory response by reducing the epidermis thickness of DNFB-induced Nc/Nga mice [21]. In this study, we demonstrated that CHD suppresses hyperkeratosis and epidermis and dermis thicknesses in AD mice.

Of note, AD symptoms are promoted by Th1 and Th2 cell cytokines [27]. In the acute phase, Th2 cells are predominant and express IL-6, IL-13, and IgE; these cytokines are related to eosinophil infiltration in AD lesions [28,29,30]. However, in the chronic phase, Th1 cells are predominant and express IFN-γ and TNF-α [28]. Importantly, Th2 cells promote the proliferation of IgE-producing B cells [10]. Excessive IgE molecules bind to FcεRI, an IgE receptor on the mast cell surface, and activate mast cells, inducing the release of inflammatory mediators, such as histamine, TNF-α, and IL-1β [10,31]. In a previous study, it was reported that *Naju Jjok* (Chung-Dae, *Polygonum tinctorium*) had anti-AD effects by inhibiting TARC, TSLP, and inflammatory cytokine expression in the dorsal tissues of DNFB-induced Nc/Nga mice [21]. Our study revealed that CHD significantly decreased the DNCB-induced high serum levels of IgE and TNF-α in the AD mice. In addition, CHD significantly reduced the high concentration levels of TNF-α, IL-6, and IL-13 in the dorsal tissues of DNCB-induced mice. Therefore, CHD relieved the severity of the clinical symptoms of AD by inhibiting the IgE and inflammatory cytokine expression levels in the serum and dorsal tissues, respectively, and by effectively reducing the dermis and epidermis thicknesses. 

Eosinophil and mast cell infiltration in the AD lesions via the Th2 cytokines is another important feature of AD [32]. The Th2 cells migrate to and damage inflamed skin sites and recruit inflammatory cells, such as mast cells and eosinophils, to impair the function of the skin barrier [33]. Activated mast cells are well established as a key cellular immune response in certain bacterial infections, hypersensitivity, and allergic disorders [34,35]. Mast cells express inflammatory mediators that stimulate the AD lesions [36]. Similarly, eosinophils are immune cells that are inflammatory mediators. They also regulate pruritus in AD [37,38]. Our results demonstrate that CHD inhibited the infiltration of mast cells and eosinophils in the dermis of DNCB-induced AD mice. Thus, we deduce that CHD inhibits immune cell infiltration in the dermis by suppressing IgE and Th2 cytokine productions.

Keratinocytes are the primary defense against bacterial and viral invasions. They are primarily used to generate several inflammatory cytokines and chemokines in skin immune response experiments [39]. Chemokines are low-molecular proteins that are divided into subfamilies based on their structure and functions: C, CC, CXC, and CX3C [40]. These chemokines enhance the inflammatory response by promoting the infiltration of leukocytes into the inflammatory site [41]. The representative Th2 chemokines TARC/CCL17 and MDC/CCL22 are produced by CD4+ T cells and induce the migration and invasion of Th2 cells into the inflammatory sites [42]. In addition, it is an important molecule because of its high concentration in the serum of patients with AD [43]. The RANTES/CCL5 chemokine is involved in the migration and activation of T cells and eosinophils and promotes inflammation by inducing adhesion of lymphocytes to the surface of endothelial cells [44]. Furthermore, MCP-1 modulates the effect of endothelial cell adhesion molecules. Activated endothelial cells induce the activity of ICAM1. This molecule promotes the migration of eosinophils, monocytes, and neutrophils during chronic allergic diseases [45,46]. The MIP-3α/CCL20 chemokine is crucial for innate and acquired immune responses because of its antibacterial activity and because it induces Langerhans cells to migrate to the skin [47,48,49]. In this study, CHD decreased the expression of TARC/CCL17, MDC/CCL22, RANTES/CCL5, MCP-1, and MIP-3α/CCL20 in TNF-α- and IFN-γ-stimulated HaCaT cells. Thus, this observation reiterates the possibility of CHD as an herbal medicine for AD treatment via suppression of pro-inflammatory chemokine production. 

The MAPK pathway comprises ERK1/2, JNK, and p38, and it is essential for the development of an inflammatory response [50]. Moreover, MAPK is a key regulator of inflammatory chemokines and cellular processes, such as cell proliferation, differentiation, and apoptosis [51,52]. In addition, it inhibits the activity of NF-κB [53,54]. The key transcription factor NF-κB regulates the transcription of genes involved in an inflammatory response [55]. Under normal physiological conditions, NF-κB p65 is bound to IκB-α. However, stimulation by cytokines, such as TNF-α/IFN-γ, phosphorylates and degrades IκB-α, resulting in translocation of NF-κB p65 to the nucleus [56]. This translocated NF-κB induces expression of inflammation-related genes in the nucleus [56]. In this study, CHD inhibited the phosphorylation of p-ERK1/2 and p-p38 and the expression of NF-κB in the dorsal skin tissues of DNCB-induced AD mice. It also blocked TNF-α/IFN-γ-induced nuclear translocation of NF-κB p65 in HaCaT cells. Our results showed that CHD regulates inflammatory chemokines by inhibiting the activation of the MAPK/NF-κB pathway. 

To find out which components contribute to the effect of atopic treatment, we performed HPLC analysis. As a result, two compounds were detected in CHD water extract and these compounds were identified indigo and indirubin. According to Pharmacopeia of the People’s Republic of China, indigo and indirubin were marker compounds of quality control of CHD [20]. Indigo increases the expression of IL-10 and IL-22 [57] and indirubin acts as an anti-inflammatory effector by decreasing T cell function and inhibiting the activation of Jak3/Stat3 pathways [58]. Based on use of treating various diseases from ancient times and the results of reported research, the effect of atopic treatment of CHD water extract was contributed including indigo and indirubin. We will confirm the atopic dermatitis effect of each component for further study.

## 4. Materials and Methods

### 4.1. AD Mice Model and Drug Treatment

**Figure 9:**
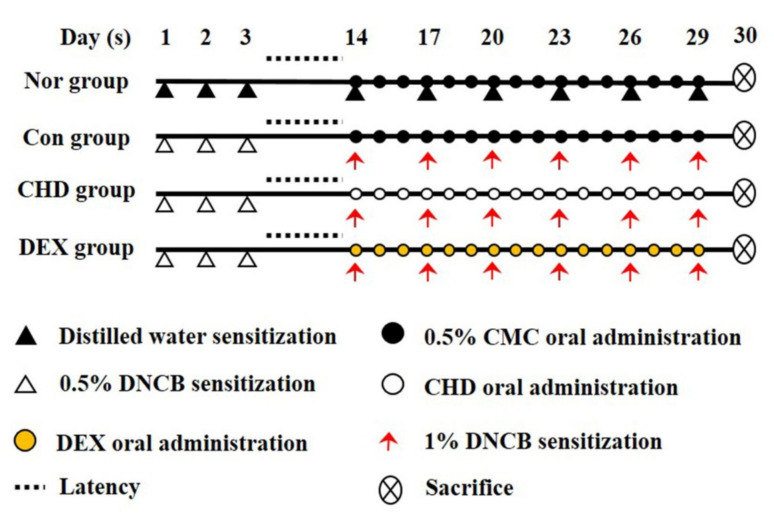
Schematic representation of the study design.

All animal experiments were approved by the Korea Institute of Oriental Medicine Animal Protection and Use Committee (no. 21-044). We obtained male 6-week-old BALB/c mice (18–20 g) from Samtako BioKorea (Osan, Korea). All animals were given food and water ad libitum and were kept at the temperature 22.5 ± 0.5 °C and the humidity 42.6 ± 1.7%. We divided these mice into five groups: normal (vehicle, *n* = 10), control (DNCB sensitive, *n* = 10), CHD 100 (DNCB + 100 mg/kg CHD, *n* = 10), CHD 200 (DNCB + 200 mg/kg CHD, *n* = 10), and DEX (dexamethasone; DNCB + 1 mg/kg DEX; positive control, *n* = 10). We anesthetized the mice by intraperitoneally injecting them with 400–500 μL of avertin (2,2,2-tribromoethanol) diluted in 2-methyl-2-butanol; this dose was based on 25 g of mice and we started with a concentration of 250 mg/kg. The dorsal skins of the mice were shaved one day before the experiment. We first sensitized the mice to DNCB by applying 200 μL of 0.5% DNCB (dissolved in a 3:1 mixture of acetone and olive oil) to their dorsal skins for three days. Subsequently, we re-sensitized them by applying 200 μL of 1% DNCB to their dorsal skins for three days. Thus, the mice were sensitized to DNCB once every 3 days, for a total of 6 times. Thereafter, we dissolved 200 μL of CHD and DEX in 0.5% of carboxymethylcellulose (CMC) for 2 h and orally administered this solution to mice belonging to the CHD treatment and the DEX groups, respectively. The normal and control groups were orally administered with 200 μL of 0.5% CMC (Figure 9). Before oral administration, this CHD and DEX were filtered through a 0.2 μm PETE syringe (Whatman, Piscataway, NJ, USA) filter before proceeding.

### 4.2. Histological Analysis

Once the mice were sacrificed, we fixed their dorsal skins with 10% neutral buffered formalin at room temperature for 24 h. We outsourced the fixed samples to a commercial company (Garam Meditech, Korea) to conduct H&E and toluidine blue staining. Eosinophils and mast cells that infiltrated into the dermis were examined under a light microscope at 400× magnification under five fields/section and 200× magnification under three fields/section, respectively. We also analyzed the epidermal and dermal thicknesses of the mice under the light microscope (100× magnification, three fields/section). Skin thickness and inflammatory cell count were measured using the ImageJ software (version 1.46; Madison, WI, USA, National Institutes of Health).

### 4.3. Preparation of CHD and Cell Culture

We dissolved the CHD powder in distilled water and centrifuged the solution at 13,000 rpm for 10 min at 4 °C. The supernatant was transferred to a clean tube and diluted with distilled water to obtain a stock concentration. Meanwhile, we dissolved the pellet in 100% DMSO and centrifuged it at 14,000 rpm for 10 min at 4 °C. These solutions were stored at −20 °C until further use.

The human keratinocyte cell line HaCaT was provided by Dr. Miseon Won, Korea Research Institute of Bioscience and Biotechnology. These cells were cultured in the Dulbecco’s modified Eagle’s medium supplemented with 10% fetal bovine serum and 1% penicillin/streptomycin at 37 °C in a humidified atmosphere of 5% CO_2_. Cells from the third passage were used for the experiments.

### 4.4. Enzyme-Linked Immunosorbent Assay (ELISA)

To perform this ex vivo assay, we collected blood samples from the anesthetized mice by cardiac puncture and separated the serum from the blood by centrifuging the samples at 2000 rpm for 10 min. Subsequently, we determined the serum IgE and TNF-α levels using a mouse IgE and TNF-α ELISA kit, according to the manufacturer’s instructions. Next, we incubated 8 × 10^4^ HaCaT cells/well in a 24-well plate for 24 h. These cells were pretreated with 50, 100, and 200 μg/mL concentrations of CHD for 1 h and stimulated with 10 ng/mL of TNF-α/IFN-γ for 24 h at 37 °C in a 5% CO_2_ atmosphere. Subsequently, we measured the concentrations of the chemokines RANTES, TARC, MDC, MCP-1, MIP-3α, and ICAM1 in the culture medium using an ELISA kit. All experiments were performed as per the manufacturer’s protocol. 

### 4.5. Quantitative Real-Time Polymerase Chain Reaction (PCR) Analysis 

For this ex vivo experiment, we extracted total RNA from the dorsal skin samples using the TRIzol reagent (Invitrogen Life Technologies, Carlsbad, CA, USA) according to the manufacturer’s instructions. Meanwhile, we incubated 4 × 10^5^ HaCaT cells/well in a 6-well plate for 24 h. We pretreated them with 50, 100, and 200 μg/mL concentrations of CHD for 1 h. We also stimulated them with 10 ng/mL of TNF-α or IFN-γ for 24 h at 37 °C in a 5% CO_2_ atmosphere. Thereafter, we extracted total RNA from these cells using the TRIzol reagent as per the manufacturer’s instructions. Total RNA was quantified using the Nanodrop 2000 spectrophotometer (Thermo Fisher Scientific, Waltham, MA, USA). We synthesized cDNA using 1 µg of total RNA as the template (Bioneer, Daejeon, Korea). Real-time RT-PCR was performed according to the manufacturer’s protocol (Bioneer, Daejeon, Korea). The PCR conditions were as follows: initial denaturation at 95 °C for 5 s, followed by 40 cycles of primer annealing at 62.5 °C for 30 s. The target genes were quantified by the 2^−ΔΔCT^ method. The expression level of each target gene was quantified using the housekeeping gene GAPDH. The primer sequences are listed in Table 2. 

### 4.6. Western Blot Analysis

For nuclear fractionation, we lysed frozen mice skin tissues using the NE-PER™ Nuclear and Cytoplasmic Extraction Reagents (cat. no. 78835, Thermo Fisher Scientific Inc., Waltham, MA, USA). To extract total protein, we lysed the tissues using the T-PER™ Tissue Protein Extraction Reagent and Extraction Buffer (cat. no. 78510, Thermo Fisher Scientific Inc., Waltham, MA, USA). The proteins were obtained by centrifugation of the lysate at 12,000 rpm at 4 °C for 10 min. The protein yield was quantified to be 30 μg, and the proteins were separated by SDS-PAGE and transferred onto a nitrocellulose membrane. The membrane was blocked with 5% skimmed milk or 3% bovine serum albumin (BSA) at room temperature for 1 h. The membrane was incubated overnight with the primary antibodies p-ERK/t-ERK, p-P38/t-P38, and NF-κB/Lamin B1 (diluted 1:1000 in 3% BSA) at 4 °C. Subsequently, the membrane was incubated with the secondary antibody at room temperature for 1 h. Bands of specific proteins were by chemiluminescence detection and semi-quantified using the ImageJ software.

### 4.7. Immunocytochemical Analysis 

We incubated the HaCaT cells with CHD (50, 100, and 200 µg/mL) in confocal dishes for 1 h and stimulated them with TNF-α/IFN-γ (10 ng/mL each) for 20 min. This was performed according to a previously described method [59]. The cells were subsequently fixed with 4% paraformaldehyde and incubated overnight with NF-κB p65 antibodies (cat. no. 8242; Cell Signaling Technology, Inc., Danvers, MA, USA) at 4 °C. Thereafter, they were incubated with Alexa-Fluor-488 secondary antibodies (cat. no. A11001; Thermo Fisher Scientific Inc., Waltham, MA, USA) at room temperature for 20 min. Nuclei were stained with DAPI (40,6-diamidino-2-phenylindole, cat. no. 8417; Sigma-Aldrich Inc., St. Louis, MO, USA) at room temperature for 10 min. The cells were visualized under a confocal microscope (FV3000 FLUOVIEW, Olympus, Tokyo, Japan).

### 4.8. CHD Sample Preparation and High-Performance Liquid Chromatography with Diode-Array Detection (HPLC-DAD)

We dissolved the two marker compounds indigo and indirubin in DMSO at each concentration and added acetonitrile to make a stock solution and diluted it 10 times. Next, we weighed the watered CHD extract and dissolved 10 mg/mL of it in DMSO and acetonitrile at a ratio of 1:1. Subsequently, we filtered this CHD extract and each of the marker compound solution through a 0.2 μm PETE membrane filter. The CHD extract and marker compound solutions were stored at 4 °C until further use. Meanwhile, we set up the various components (column oven, binary pump, diode array UV–vis detector, and an autosampler) of the HPLC-DAD equipment Dionex UltiMate 3000 system (Dionex Corp., Sunnyvale, CA, USA). We then injected 10 µL of the CHD extract and marker compound solutions into the HPLC column (250 × 4.6 mm, 5 μm X bridge C18 column with 25 °C temperature) at a flow rate of 1.0 mL/min. The CHD-HPLC spectrum was detected under the following conditions: HPLC solvent A, 0.1% formic acid and HPLC solvent B, acetonitrile. We applied the following eluted gradient method: 0–15 min, 55–80% A and 15–25 min, 80–100% B. The UV detector was set at 286 nm. The peak area was calculated using the Chromeleon 7 software. Notably, all the samples were injected in triplicates in the same conditions.

### 4.9. Statistical Analysis

All experiments were repeated at least three times. Statistical analysis was performed using GraphPad Prism Software (version 5.01; GraphPad Software, Inc., San Diego, CA, USA). Data are presented as mean ± SEM assessed using Student’s *t*-test or analysis of variance (one-way ANOVA, Dunnett, control comparison). *p* < 0.05 was considered to indicate a statistically significant difference. 

## 5. Conclusions

We demonstrated that CHD effectively treated AD in vivo by inhibiting the expression of inflammatory cytokines and the phosphorylation of MAPK and NF-κB. In addition, it inhibited chemokine production and NF-κB p65 nuclear translocation in TNF-α- and IFN-γ-induced HaCaT cells. Thus, we demonstrate the potential of CHD as a prophylactic agent in treating AD by inhibiting several inflammatory mediators in vivo and in vitro.

## Figures and Tables

**Table 1:** Calibration curve of marker compounds.

Compound	Range (ug/mL, ppm)	Regression Equation	r^2^	LOD (μg/mL)	LOQ (μg/mL)
Indigo	10.0~80.0	y = 0.3334x − 0.5198	0.9958	0.0164	0.0499
Indirubin	2.0~16.0	y = 1.7955x + 0.2972	0.9997	0.0030	0.0091

LOD = 3.3 × σ/S. LOQ = 10 × σ/S. σ is the standard deviation of the intercept from the regression equation and S is the slope of the calibration curve.

**Table 2:** Primer sets for real-time RT-PCR.

Name	Forward	Reverse
h-RANTES	5′ GATGCCAAAG AGAGAGGGAC 3′	5′ AAATTTGTGT AAGTTCAGGT 3′
h-TARC	5′ CTGCACACAG AGACTCCCTC 3′	5′ CTGGTACCAC GTCTTCAGCT 3′
h-MDC	5′ GAAACACTTC TACTGGACCT 3′	5′ CAGGGAGGTA GGGCTCCTGA 3′
h-GAPDH	5′ TCAAGGCTGA GAACGGGAAG 3′	5′ TGGACTCCAC GACGTACTCA 3′
m-TNF-α	5′ ATGAGCACAG AAAGCATGAT 3′	5′ TACAGGCTTG TCACTCGAAT 3′
m-IL-6	5′ TTCCATCCAG TTGCCTTCTT 3′	5′ ATTTCCACGA TTTCCCAGAG 3′
m-IL-13	5′ CATCTCCAAT TGCAATGCCA 3′	5′ GCCCAGGGAT GGTCTCTCCT 3′
m-GAPDH	5′ AACGACCCCT TCATTGAC 3′	5′ TCCACGACAT ACTCAGCAC 3′

h-, human; m-, mouse; RANTES, regulated on activation, normal T cell expressed and secreted, CCL5; TARC, thymus and activation-regulated chemokine, CCL17; MDC, macrophage-derived chemokine, CCL22.

## Data Availability

Not applicable.

## Supplementary Materials

The following supporting information can be downloaded at: https://www.mdpi.com/article/10.3390/ijms23010553/s1.
