# Supplementary material for: Indigo Pulverata Levis (Chung-Dae, Persicaria tinctoria) Alleviates Atopic Dermatitis-like Inflammatory Responses In Vivo and In Vitro
Source: Int J Mol Sci. 2022 Jan 5;23(1):553. doi: 10.3390/ijms23010553 (PMC8745452; doi:10.3390/ijms23010553)
Supplement: Supplementary file 1 [file ijms-23-00553-s001.zip › ijms-1510016-supplementary.pdf]

Method

S1. Cell Viability Assay

HaCaT cells were seeded in a 96 well plate, with 8x10[3] cells/well. After 24 h, CHD (50, 100 and 200 μg/mL) was added to the medium for 24 h. Then, 20 μL of the EZ-Cytox solution were added to each well. After incubation for 2 h at 37 °C, the optical density was measured using an ELISA (Infinite M200, Tecan, Männedorf, Switzerland) at an optical density of 450 nm.

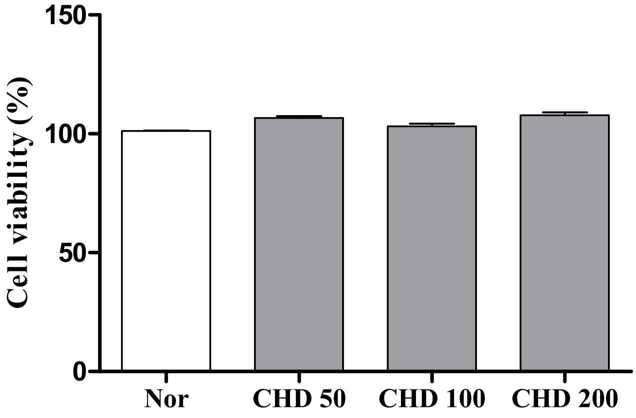

Figure S1. Effects of CHD on cytotoxicity of TNF-α/IFN-γ-stimulated HaCaT cells. The cytotoxicity of CHD was measured using a Spectrophotometer.
